# Molecular Detection of Zoonotic and Non-Zoonotic Pathogens from Wild Boars and Their Ticks in the Corsican Wetlands

**DOI:** 10.3390/pathogens10121643

**Published:** 2021-12-20

**Authors:** Baptiste Defaye, Sara Moutailler, Christian Pietri, Clemence Galon, Sébastien Grech-Angelini, Vanina Pasqualini, Yann Quilichini

**Affiliations:** 1 UMR CNRS SPE 6134, Université de Corse Pascal Paoli, 20250 Corte, France; pasqualini_v@univ-corse.fr; 2 Anses, INRAE, Ecole Nationale Vétérinaire d’Alfort, UMR BIPAR, Laboratoire de Santé Animale, 94700 Maisons-Alfort, France; sara.moutailler@anses.fr (S.M.); clemence.galon@anses.fr (C.G.); 3 Fédération Départementale des Chasseurs de Haute-Corse, St-Joseph, 20600 Bastia, France; fdc2b.pietri@orange.fr; 4 Groupement Technique Vétérinaire de Corse, 20240 Ghisonaccia, France; grech.angelini@gtvcorse.fr

**Keywords:** *Anaplasma*, *Babesia*, *Rickettsia*, Corsica, tick-borne pathogens, ticks, wild boars, wetlands

## Abstract

Corsica is the main French island in the Mediterranean Sea and has high levels of human and animal population movement. Among the local animal species, the wild boar is highly prevalent in the Corsican landscape and in the island’s traditions. Wild boars are the most commonly hunted animals on this island, and can be responsible for the transmission and circulation of pathogens and their vectors. In this study, wild boar samples and ticks were collected in 17 municipalities near wetlands on the Corsican coast. A total of 158 hunted wild boars were sampled (523 samples). Of these samples, 113 were ticks: 96.4% were *Dermacentor marginatus*, and the remainder were *Hyalomma marginatum*, *Hyalomma scupense* and *Rhipicephalus sanguineus* s.l. Of the wild boar samples, only three blood samples were found to be positive for *Babesia* spp. Of the tick samples, 90 were found to be positive for tick-borne pathogens (rickettsial species). These results confirm the importance of the wild boar as a host for ticks carrying diseases such as rickettsiosis near wetlands and recreational sites. Our findings also show that the wild boar is a potential carrier of babesiosis in Corsica, a pathogen detected for the first time in wild boars on the island.

## 1. Introduction

The wild boar (*Sus scrofa*) is one of the most widely distributed ungulates in the world, present on all continents except Antarctica [1]. The distribution of this species has increased over the years through increased farming or reintroduction or introduction for hunting, and due to vigorous reproduction and the animals’ adaptable feeding habits [2,3,4]. Wild boars can represent a major hazard via direct or indirect interaction with many different species, including domestic pigs, livestock [5,6,7], and humans. These interactions can increase the risk of transmission of pathogens [8,9,10,11]. Currently in the Mediterranean region, the wild boar is known to act as a reservoir for many disease-causing agents through interactions with many different populations and in multiple landscapes, including in Corsica (western Mediterranean) [10,12].

Wild boars live close to water, especially wetlands, mainly because of their diet [13]. Wetlands are areas that are key to human activities and where there are high levels of interactions with animals. Wetlands represent one of the main watering and resting areas for both animals and humans [14,15]. These habitats are important for public health given their role in vector proliferation and pathogen transmission [16,17,18]. Risks are mostly related to the animal species found in the wetland of interest. Depending on the ecosystem, the diversity and the prevalence of ectoparasite communities and pathogens can change [19]. 

Worldwide, the wild boar is host to many types of pathogens, such as food-borne, tick-borne, and mosquito-borne pathogens [9,20,21]. Across Europe, *S. scrofa* can carry a large spectrum of tick-borne pathogens, including *Anaplasma* spp., *Rickettsia* spp., *Ehrlichia* spp., *Theileria* spp., *Borrelia* spp., *Babesia* spp., and tick-borne encephalitis virus [8,9,11,20,22]. It has been confirmed in Corsica that *S. scrofa* is an important host of many diseases and parasites [12,23,24]. On this island, wild boar hunting is a very long-standing tradition that is still very popular, with over 26,000 wild boars hunted in 2017 [25]. One of the main routes of pathogen transmission from wild boars to humans is via food, especially in Corsica, where wild boar meat is widely consumed. Pathogens such as the hepatitis E virus are highly prevalent in the Corsican population (>60% of blood donors) and in wild boars. The population may also be exposed to *Trichinella britovi* and *Echinococcus canadensis* [12,26,27,28]. Another route of transmission is direct or indirect interactions that cover different kinds of pathogens: viruses such as the Aujeszky’s disease virus, bacteria such as *Mycobacterium bovis*, and parasites such as *Toxoplasma gondii* have been found in wild boars in Corsica [12,29,30]. Transmission may also occur through vectors, especially ticks. The five tick species found on wild boars in Corsica are known to be vectors of different kinds of disease [31]. To date, *Rickettsia* from the spotted fever group (*Rickettsia slovaca* and *Rickettsia aeschlimannii*), *Rickettsia raoultii*, *Rickettsia conorii* (the agent of Mediterranean spotted fever), *Anaplasma* spp. and *Bartonella* spp. are the only tick-borne pathogens recorded from ticks collected from wild boars in Corsica [32,33,34]. The wild boar may play an important role in the transmission of these pathogens [9,35]. The species serves as a maintenance host and contributes to distribution by spreading ticks as part of its numerous interactions with other species [6,9]. Even though wild boars can play a major role in pathogen transmission and ectoparasite spreading, few studies have focused on the extensive detection of pathogens in both animals and vectors. Detecting pathogens simultaneously in animals and in the ectoparasites they carry makes it possible to observe animal-borne and ectoparasite-borne pathogens in the same area.

Among potential vectors, ticks (Acarida) rank first for veterinary vector-borne pathogens and second for human disease, just after mosquitoes. Ticks are involved in the transmission, circulation and maintenance of pathogens between different groups of animals worldwide [36,37]. Tick-borne pathogens are maintained in a stable, natural cycle involving their incidental host and reservoir (such as animals) and accidental host (such as humans) by blood-feeding by vectors [38]. In this way, ticks can transmit a range of bacteria, viruses, and parasites. These tick-borne pathogens are known to be both pathogens of veterinary importance and zoonotic pathogens harmful to humans [39]. Many different animals are involved as reservoirs. In European countries, there are a diversity of important reservoir groups, such as birds, rodents, domestic and wild ungulates, such as the wild boar [40]. In four surveys of tick fauna, 10 species were found in Corsica from rodent pets and domestic and wild ungulates: *Amblyomma variegatum* (sporadically), *Ixodes ricinus*, *Rhipicephalus bursa*, *Rhipicephalus sanguineus* s.l., *Rhipicephalus annulatus*, *Hyalomma marginatum*, *Haemaphysalis sulcata*, *Haemaphysalis punctata*, *Hyalomma scupense* and *Dermacentor marginatus* [31,32,41,42,43]. Five species have been found in wild boars. Out of the five species, *D. marginatus* was the most common, constituting >80% of the collected ticks. The other species are *Rh. bursa*, *Rh. sanguineus* s.l., *Hy. marginatum* and *I. ricinus*.

The aims of this study were to (i) determine the main tick species of wild boars in Corsican wetlands, and (ii) obtain an overview of the potential presence of a wide range of zoonotic and non-zoonotic pathogens in wild boars and their ticks in the Corsican wetlands. 

For this purpose, we collected tissue samples and ticks from wild boar from the Corsican wetlands, and we focused the screening on the detection of 34 species and 11 genera of bacteria, viruses, and parasites expected to be present and belonging to the main tick-borne pathogens or non-tick-borne pathogens with an considerable impact on animal and human health, including: African swine fever virus, *Anaplasma* spp., *Anaplasma marginale*, *Anaplasma phagocytophilum*, Aujeszky’s virus, *Babesia bigemina*, *Babesia bovis*, *Babesia caballi*, *Babesia divergens*, *Babesia microti*, *Babesia ovis*, *Babesia vogeli*, *Babesia venatorum*, *Bartonella* spp., *Bartonella henselae*, *Bartonella quintana*, *Borrelia afzelii*, *Borrelia burgdorferi* spp., *Borrelia burgdorferi sensu stricto*, *Borrelia lusitaniae*, *Borrelia miyamotoi*, *Borrelia spielmanii*, *Chlamydia psittaci*, *Coxiella burnetii*, *Ehrlichia* spp., *Ehrlichia ruminatum*¸ *Haemoproteus* spp., *Hepatozoon* spp., *Leishmania* spp., *Leishmania infantum*, *Leptospira* spp., *Leucocytozoon* spp., *Neoehrlichia mikurensis*, *Plasmodium* spp., *Rickettsia* spp., *R. aeschlimannii*, *R. conorii*, *Rickettsia helvetica*, *Rickettsia massiliae*, *Rickettsia monacensis*, *R. slovaca*, *Theileria annulata*, *Theileria equi*, *T. gondii* and *Trypanosoma* spp by means of microfluidic real-time PCR.

## 2. Results

### 2.1. Tick Identification

Of the 158 wild boar samples from 17 municipalities, a total of 113 ticks were collected from 32 wild boars from nine areas close to coastal wetlands (Table 1). Overall, 96.4% of them were *D. marginatus*; the remainder belonged to three species: *Hy. marginatum*, *Hy. scupense* and *Rh. sanguineus* s.l.. They were all adults, with 53.6% being males and 46.4% being fully and partially engorged females.

### 2.2. Detection of Tick-Borne Pathogen DNA in Wild Boar Samples

**Figure 1:**
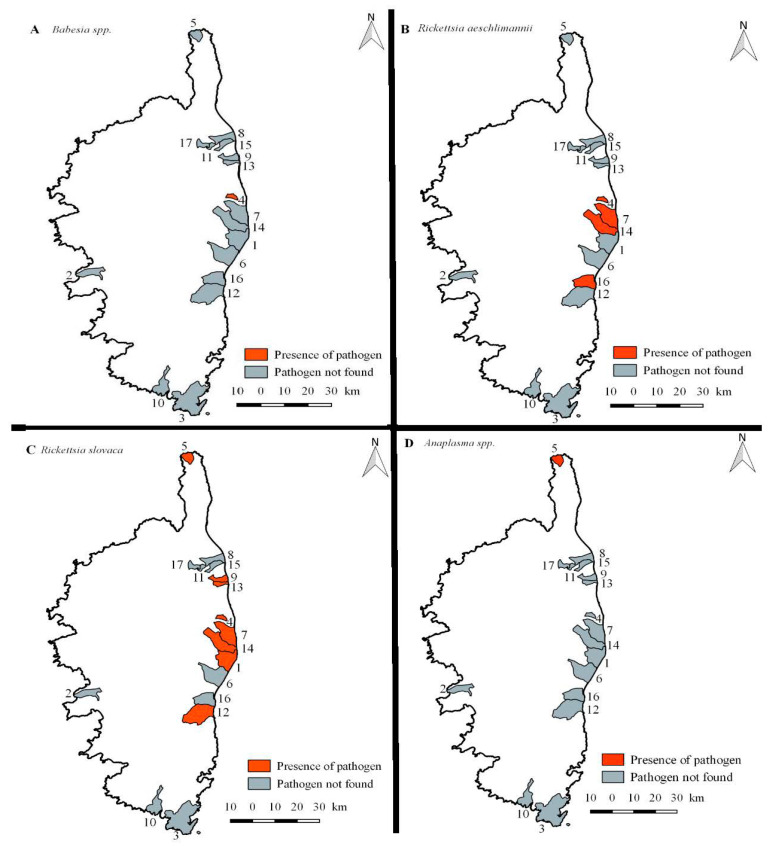
Map of Corsica showing the locations of positive samples from wild boars and ticks (1. Aléria, 2. Appietto, 3. Bonifacio, 4. Chiatra, 5. Ersa, 6. Ghisonaccia, 7. Linguizetta, 8. Luciana, 9. Penta-di-casinca, 10. Pianottoli-Caldarello, 11. Prunelli-di-Casaconi, 12. Solaro, 13. Taglio-Isolaccio, 14. Tallone, 15. Vescovato, 16. Ventiseri, 17. Volpajola).

Only three wild boar samples were found to be positive. These were three blood samples from three juvenile males from Chiatra municipality which were positive for *Babesia* spp. (Table 1 and Figure 1A). Unfortunately, the sequencing did not enable us to identify the *Babesia* species involved. This was the only pathogen found in wild boar blood samples, and no pathogens were found in the other tissue samples (spleen and liver). 

### 2.3. Detection of Tick-Borne Pathogen DNA in Ticks

Of the 113 ticks collected from wild boars, 90 were positive (79.6%) and two genera were identified. The main genus was *Rickettsia* spp., with two species. Of the 89 tick samples positive for rickettsiae, three were *Rickettsia* spp. and two species were confirmed by sequencing: *R. slovaca* and *R. aeschlimannii*.

Six ticks were positive for *R. aeschlimannii*: 2.8% of the *D. marginatus*, 50% of the *H. marginatum*, and 100% of the *Hy. scupense* and *Rh. sanguineus* s.l. (Table 1 and Figure 1B). Of the six positive samples, two were males collected on juvenile wild boars, and the other four were one female and three males collected on adult wild boars. These six samples were found to be related to the ompB sequence of *R. aeschlimannii* detected in *H. marginatum* collected from the environment in the Crimean Peninsula (GenBank: KU961544.1), with 88–98% identity to the sequence for one *D. marginatus* and more than 99% for the other five. 

A total of 80 ticks were positive for *R. slovaca*: 73.4% of the *D. marginatus* collected (Table 1 and Figure 1C). About half of the ticks were male (52.5%), and the other half female (47.5%). Most ticks were collected from adults rather that juveniles (98.8% and 1.2%) and female rather than male wild boars (68.8% and 31.8%). *R. slovaca* sequences found were related to the *R. slovaca* ompB sequence from a *Dermacentor* spp. nymph collected on a Royle’s mountain vole (GenBank: MN581993.1), with an identity between 96% and 100%, and from an *R. slovaca* citrate synthase sequence from a *D. marginatus* tick from *S. scrofa* in the south of France (GenBank: AY129301.1), with an identity between 99% and 100%.

The last tick was detected as being positive for *Anaplasma* spp. It was a male *D. marginatus* collected from a male wild boar in Ersa municipality (Table 1 and Figure 1D). Unfortunately, the sequencing of *Anaplasma* spp. in this sample was unsuccessful.

## 3. Discussion

In this paper, we report two wild boar hunting season surveys (2018–2019 and 2019–2020) in several coastal or near-coastal administrative municipalities of Corsica with the aim of identifying major non-vector pathogens, tick-borne pathogens, and their vectors.

### 3.1. Ticks on Wild Boar Population in Corsican Wetland 

Across the Mediterranean Rim, a high diversity of Ixodidae ticks is found on wild boars. Their distribution can be divided into two areas: the western side, with the highest diversity, and the eastern side, where ticks were found only in Israel and Turkey. A total of five genera were found on the western side: *Dermacentor*, *Haemaphysalis*, *Hyalomma*, *Ixodes* and *Rhipicephalus*, with a total of nine different species; and four genera on the eastern side: *Dermacentor*, *Haemaphysalis*, *Hyalomma*, and *Rhipicephalus*, with a total of seven different species (Table 2). 

Of these tick species, five are the main ticks found in various studies. The term main specie defines the species found with the highest number of tick individuals on the wild boar in each study. The most important is *D. marginatus*, which is the main tick found on wild boars in more than half of the publications in the Mediterranean Rim. This can be explained by the tropism of the adult stage mainly for wild ungulates, especially wild boars [41]. On the western side, the other main species are *Hy. marginatum* and *Hy. lusitanicum*, which mainly target livestock but can occur on wild ungulates [44]. In the Eastern part, the two other main species are *H. parva* and *Hy. detritum*, which have previously been found mainly in domestic ruminants but can also target wild ungulates [44,45]. 

**Figure 2:**
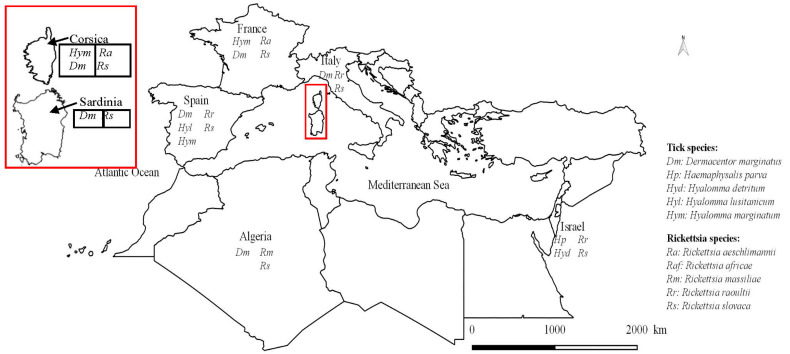
Distribution of Rickettsiae species found in ticks collected from wild boars (*Sus scrofa*) in the Mediterranean Rim (data from the literature).

In Corsica, ticks from four genera have previously been found on wild boars: *Dermacentor*, *Ixodes*, *Hyalomma* and *Rhipicephalus*. Tick belonging to the genus *Rhipicephalus* and the species *Hy. marginatum* were the main ones found on domestic ungulates and pets. On the other hand, *I. ricinus* was more commonly found on deer and pets [31,32]. Our results are consistent with those of studies performed elsewhere in the Mediterranean Rim. This can be explained by the distribution of *D. marginatus*, which is mainly found in the west Mediterranean Rim (Figure 2), due to its tropism for the wild boar, and its activity peak in autumn and winter [44]. The three other species found (*Hy. marginatum*, *Hy. scupense*, which also infest ungulates with a tropism for domestic animals, and *Rh. sanguineus* s.l., which infest both dog and ungulates in the Mediterranean Rim) have an activity peak in spring [41]. However, the tropism for the wild boar, the activity peak of *D. marginatus* and the seasonal collection of ticks (autumn and winter) can explain the difference in proportions between the *D. marginatus* found and other tick species, such as *Hyalomma* spp., which are more present in spring and summer. Seasonal surveys of ticks on wild boars from wetlands could be useful to determine the circulation of tick-borne pathogens linked to wild boars, and the threat to animals and humans.

**Table 2:** Tick-borne pathogens detected in the wild boar (*Sus scrofa*) and its ticks in the Mediterranean Rim.

Area (Island)	Main Tick Species	Other Tick Species	Pathogens Found in Ticks (Bacterium ^b^, Parasite ^p^ and Virus ^v^)	Pathogens Found in Wild Boars (Bacterium ^b^, Parasite ^p^ and Virus ^v^)	References
Algeria	*D. marginatus*	*H. punctata, Rh. sanguineus*	*R. massiliae*^b^, *R. slovaca*^b^	Not found	[46]
Algeria			Not screened	*R. slovaca* ^b^	[47]
France (Corsica)	*D. marginatus*	*Hy. marginatum, H.scupense*, *I. ricinus*	*A. phagocytophilum*^b^*, R.* spp. ^b^, *R. aeschlimannii* ^b^, *R. slovaca* ^b^	*Babesia* spp. ^b^	Present study
France (Corsica)	*D.marginatus*	*Hy. marginatum, I. ricinus*, *Rh. bursa*	*R. aeschlimannii*^b^, *R. slovaca*^b^	Not screened	[32]
France (Corsica)	*D. marginatus*	*Hy. marginatum, I. ricinus*, *Rh. bursa, Rh. sanguineus*	*A. phagocytophilum*^b^, *Ba. henselae*^b^, *R. aeschlimannii*^b^, *R. slovaca^b^*	Not screened	[33]
France (Corsica)	*Hy. marginatum*		*R. aeschlimannii* ^b^	Not screened	[48]
France			Not screened	Tick-borne encephalitis virus ^v^, Louping ill ^v^	[49]
France			Not screened	*A. phagocytophilum* ^b^	[50]
France	*D. marginatus*		*Rickettsia* spp. ^b^, *R. slovaca ^b^*	Not search	[51]
Israel	*H. parva*	*H. aleri, Hyalomma* spp., *Rh. turanicus*	*R. massiliae* ^b^	Not screened	[52]
Israel	*Hy. detritum*		*R. africae* ^b^	Not screened	[53]
Italy			Not screened	*A. phagocytophilum*^b^*, Bo. burgdoferi* s.l. ^b^	[54]
Italy	*D. marginatus*		*R. raoultii*^b^, *R. slovaca*^b^	Not screened	[55]
Italy (Sardinia)	*D. marginatus*		*R.slovaca* ^b^	Not screened	[56]
Italy (Sardinia)	*D. marginatus*		*R.* spp. ^b^	Not screened	[57]
Italy	*D. marginatus*		*R. slovaca* ^b^	Not screened	[58]
Italy	*D. marginatus*		*E. canis* ^b^	Not screened	[59]
Italy			Not screened	*B.bigemina*^p^, *Theileria* spp. ^p^	[60]
Slovenia			Not screened	*A. phagocytophilum* ^b^	[61]
Slovenia			Not screened	*A. phagocytophilum* ^b^	[62]
Spain	*Hy. marginatum*	*D. marginatus, Rh. bursa*	*A. marginale ^b^*, *A. phagocytophilum ^b^, Ehrlichia* spp. ^b^, *Piroplasmid* ^p^ *Rickettsia* spp. ^b^, *R. slovaca* ^b^	Not screened	[22]
Spain	*D. marginatus*	*D. reticulatus, Rh. bursa*	Not found	*C. burnetii* ^b^	[63]
Spain	*D. marginatus*		*R.slovaca* ^b^	Not screened	[64]
Spain	*D. marginatus*		*R. slovaca* ^b^	Not screened	[65]
Spain	*Hy. lusitanicum*	*D. marginatus*	*R. raoultii*^b^, *R. slovaca*^b^	Not screened	[66]
Turkey	*D. marginatus*	*H. parva, Hy. excavatum, Hy. marginatum, Rh. turanicus*	*B. crassa*^p^, *B.occultans*^p^, *B. rossi*^p^	Not found	[67]

### 3.2. Pathogens Detected in Wild Boar and Their Ticks in the Corsican Wetlands

Another objective of our study was to overview the potential presence of a wide range of pathogens in wild boars and their ticks in the Corsican wetlands. Concerning wild boars in the Mediterranean Rim, most pathogens were found on ticks. We found only one article focusing on Algeria, and another on Turkey [46,67], in which the pathogens were searched for in both ticks and wild boars. Screening for pathogens in both ticks and hosts enables us to research tick-borne pathogens in ticks and non-vector pathogens in wild boars from the Corsican wetlands. 

#### 3.2.1. *Babesia* spp.

In this study, only the *Babesia* genus was found in three wild boar blood samples. All of the remaining tissue and blood samples were negative. This genus is composed of an intracellular protozoan parasite from the piroplasmid group. It targets erythrocyte cells and is transmitted by hard ticks. It mainly infects livestock and pets, but can also be found in wild ungulates and rodents [60,68]. Parasites of the *Babesia* genus can be responsible for moderate symptoms such as myalgia, headache, anorexia, as well as severe symptoms such as pulmonary edema, organ dysfunction syndrome and coma [69]. The zoonotic species are mainly transmitted by ticks from the *Ixodes* genus and are primarily *B. divergens*, *B. duncani*, *B. microti*, and *B. venatorum*. Their primary reservoir is often rodents, which contaminate immature tick stages, and the pathogens can be transmitted to ungulates during the adult stage [69]. In the Mediterranean Rim, one species of the *Babesia* genus (*B. bigemina*) has already been detected in wild boar in Italy, with a prevalence of 4.67% [60], and no *Babesia* was found in ticks. In Corsica, *B. bigemina* was found in *I. ricinus* collected from cattle and goats; *B. ovis* was found only in *I. ricinus* collected from cattle for domestic animals, and from mouflons for wild ungulates [33]. Our findings are the first possible report of *Babesia* spp. DNA in wild boars from Corsica; however, the sequences could not be obtained, so the presence of *Babesia* spp. in wild boar still requires confirmation. 

#### 3.2.2. *Rickettsia* spp.

The *Rickettsia* genus was the most detected in the tick samples; however, *Rickettsia* DNA was not detected in the tissue samples of wild boars. This genus is composed of three groups: the spotted fever group (SFG), the typhus group and the transitional group. The SFG is the only group transmitted exclusively by ticks, which have both the role of vector and reservoir thanks to their transstadial and transovarial transmission of the pathogens. It is composed of many zoonotic agents with diverse pathogenicity levels, but most of these species can be a threat for human health, with symptoms ranging from a cutaneous rash to lymphadenopathy, for example [70,71]. In Corsica, even if the exact impact of *Rickettsia* species is still unknown, diverse species were already found in animals (*R. aeschlimannii, R. africae*, *R. felis, R. helvetica*, *R. massiliae* and *R. slovaca* [32,33,43,48]), and a previous sero-epidemiological study showed the exposition of 4.8% of the people to these pathogens in “Corse du Sud” [72]. 

In our study, the main *Rickettsia* species detected was *R. slovaca*, found in 73.4% of collected *D. marginatus*. *R. slovaca* is a tick-borne rickettsia species from the spotted fever group, mainly transmitted by *D. marginatus* [73]. This rickettsia species is mainly known for causing tick-borne lymphadenopathy in humans [74]. It was first isolated in a *D.* marginatus tick in Slovakia in 1968. In Europe, *R. slovaca* has already been detected in several countries in Eastern Europe, including Slovakia, Romania, and Bulgaria, as well as in southern Europe, including France, Spain, Portugal and Italy, mainly with the involvement of *D. marginatus* and *D. reticulatus* [74,75,76,77]. In the Mediterranean Rim, *R. slovaca* is one of the most frequent tick-borne pathogens found in wild boar ticks, mainly in the north-west of the Mediterranean Rim and in Algeria. It is mainly found in *D. marginatus*, and the only detection in wild boar tissues was from the North of Algeria, with a prevalence of 5.4% in wild boar spleens [47]. Concerning Mediterranean islands, *R. slovaca* has already been found in Sardinia from *D. marginatus, Rh. bursa* and *H. marginatum* collected from wild boars, mouflons and foxes, and in *D. marginatus* collected from humans in Sicily. In Corsica, *R. slovaca* has already been detected from *D. marginatus, I. ricinus* and *Rh. bursa* from wild boars, and *D. marginatus* from cats [32,33,78,79,80]. The prevalence of *R. slovaca* (70.8%) found in our study was quite similar to that previously found in Corsica (66%) [32,33]. In this study, we show the importance of *D. marginatus* ticks in *R. slovaca*’s possible presence near wetland areas in Corsica, where humans and animals could be at risk from this pathogen.

*R.aeschlimannii* belongs to the spotted fever group of rickettsiae, similarly to *R. slovaca*, and was first found in *Hy. marginatum* from Morocco. This disease was detected in a man who traveled to Morocco in 2000 [81,82]. Given its circulation via *Hy. marginatum*, *R. aeschlimannii* has a different distribution pattern compared to *R. slovaca*, following the distribution of *H. marginatum*, mainly in Africa. It has also been found in southern Europe and in the Mediterranean Rim. *R. aeschlimannii* has been detected in species other than *H. marginatum*, such as *Rhipicephalus* and *Ixodes* ticks [83]. As with *R. slovaca*, *R. aeschlimannii* was mainly found in ticks. Regarding the Mediterranean islands, *R. aeschlimannii* seems to follow migratory birds that carry ticks from the *Hyalomma* genus, such as *Hy. marginatum* and *Hy. rufipes*, carrying *R. aeschlimannii* in Greece and the Italian islands [84,85]. In wild boars, unlike *R. slovaca*, *R. aeschlimannii* is only found on the north-west border of the Mediterranean Rim. In Corsica, *R. aeschlimannii* was also detected in *Rhipicephalus*, *Hyalomma*, and *Ixodes* ticks collected from a wide range of domestic ungulates and wild boars [32,33]. In wetlands, *R. aeschlimannii* was detected in wild boars with a lower prevalence, but at a high prevalence in the ticks of the genus *Hyalomma* and *Rhipicephalus* (50–100%), which confirms the importance of these tick genera in *R. aeschlimannii*’s potential circulation in Corsica. On the other hand, since the pathogen was not found in the wild boar, this animal species seems not to be involved in *R. aeschlimannii*’s circulation in this area. 

#### 3.2.3. *Anaplasma* spp.

The other genus found in ticks was was *Anaplasma* spp., which belongs to the *Anaplasmataceae*, and is known to be a pathogen targeting ruminants. The members of this genus are obligatory intracellular bacteria which can be responsible of febrile illness and can cause diseases such as human granulocytic anaplasmosis. They are mainly transmitted by hard ticks of the *Ixodes* genus [86]. The confirmation of the transstadial and transovarial transmission of *Anaplasma* bacteria seems to also show the reservoir behavior of the ticks [87,88]. This genus has a worldwide distribution [89,90,91]. From ticks collected from wild boars of the Mediterranean Rim, *A. marginale* and *A. phagocytophilum* have been detected in Slovenia and Spain. This genus was also one of the most frequently detected in wild boar tissue samples, with the detection of *A. phagocytophilum* in France, Italy, and Slovenia. In the Mediterranean Rim, unlike *Rickettsia* spp., *Anaplasma* spp. was found to a moderate extent in ticks and wild boars. In Corsican ticks, *A. phagocytophilum* has already been found in ticks collected from birds, livestock, and wild boars, while *A. marginale* was detected in ticks from cattle and mouflons and *A. ovis* was detected in ticks from goats [33,92,93]. From animals in Corsica, *A. bovis*, *A. marginale*, and *A. omtjenn* were found in cattle’s blood samples and *A. ovis* was found in goats’ blood samples [33,93,94]. The observed prevalence of *Anaplasma* spp. (0.9%) was lower than the prevalence previously found in wild boars from Corsica (2%) [33]. In our study, we confirmed the presence of *Anaplasma* spp. near the Corsican wetlands. 

The results of this study confirm the importance of pathogen research in both animals and their associated tick species, in order to draw conclusions described above about the potential risks to animal and human health in the Corsican wetland. In our study, as no pathogen DNA was found in common between the wild boar and their ticks, no statement can be made regarding the transmission of these pathogens between both species. However, the methodology used was based on PCR detection and did not allow us to confirm the presence and the circulation of these pathogens. Nevertheless, these results are a first step in the characterization of the potential threat regarding those pathogens in hotspots of human and animal activity in Corsica. Obviously, serological surveys and pathogen isolations are needed to draw conclusions regarding the presence and potential circulation of these pathogens in the wild boar population of the Corsican wetlands. Future studies are needed to assess the extent of major non-vector pathogens, tick-borne pathogens and their vectors in wetland compared to other land types in the wild boar population of Corsica.

## 4. Materials and Methods

### 4.1. Animals and Sampling 

Corsica is the third largest island in the Mediterranean Sea (8722 km²). It is located 15 km north of the closest Italian territory (Sardinia) and 160 km from the coast of mainland France. It is characterized by a mild Mediterranean climate and has a huge spectrum of landscapes, such as wetlands (mainly on the coast), forests, and mountains (Monte Cintu peaks at 2706 m). Corsica is divided into two administrative departments, Haute-Corse and Corse du Sud, with a population of 322,000, which is multiplied by 10 during the tourist season, with approximately 3 million tourists, located primarily around the coastal wetlands. From autumn 2018 to winter 2020, a total of 523 samples were collected from 158 hunter-harvested wild boars in 17 municipalities close to wetlands. In all, 100 blood samples, 158 liver samples, 152 spleen samples, and 113 fully and partially engorged ticks were collected by hunters just after wild boar death during the hunting season, in compliance with current regulations. All samples were stored at −80 °C until further study. Every animal was morphometrically characterized by the hunter. Age was determined on the basis of tooth eruption patterns. The animals were classified as juveniles if they were under 12 months of age, and as adults above 12 months of age. Wetlands were divided into 4 categories: lagoon, river/river mouth, artificial lake, and temporary pool. 

### 4.2. DNA Extraction and PCR Pre-Amplification 

Ticks were washed in ethanol and distilled water and then morphologically determined to the species level using standard keys [37,38]. Prior to extraction, tissues and ticks were crushed in microtubes filled with six metal beads using a fisher brand Bead Mill 24 (Thermofisher, Waltham, MA, USA) at 5500 rpm for 20 s. DNA from ticks and tissues was extracted using a Nucleospin tissue kit (Macherey-Nagel, Düren, Germany). For the blood samples, a Nucleospin Quickpure Blood kit (Macherey-Nagel, Düren, Germany) was used, according to the manufacturer’s instructions. All the ticks were analyzed individually. 

To improve the detection of pathogen DNA, total DNA was pre-amplified using a PreAmp Master Mix, according to the manufacturer’s instructions (Fluidigm, South San Francisco, CA, USA). Prior to experiments, primers targeting all pathogens were pooled in equal volumes (200 nM each). The experiment was performed with 1 µL of PreAmp Master Mix, 1.25 µL of pooled primers mix, 1.5 µL distilled water, and 1.25 µL DNA for 5 µL final volume. The pre-amplifications were performed with one cycle at 95 °C for 2 min, 14 cycles at 95 °C for 15 s and 60 °C for 4 min. At the end of pre-amplification, samples were diluted to 1:10. Pre-amplified DNA was stored at −20 °C until further use.

### 4.3. Assay Design 

Vector-borne pathogens and their targeted genes were *Anaplasma* spp. (16S), *A. marginale* (Msp1b), *A. phagocytophilum* (Msp2), *Borrelia* spp. (23S), *Bo. burgdorferi* s.s. (rpoB), *Bo. afzelii* (flagellin), *Bo. miyamotoi* (glpQ), *Bo. lusitaniae* (rpoB), *Bo. spielmanii* (flagellin), *Rickettsia* spp. (gltA), *R. slovaca* (23S-5S ITS), *R. helvetica* (23S-5S ITS), *R. aeschlimannii* (23S-5S ITS), *R. massiliae* (23S-5S ITS), *R. conorii* (23S-5S ITS), *C. burnetii* (idc, IS1111), *N. mikurensis* (ARN16S), *Bartonella* spp. (ssrA), *Ba. henselae* (pap31), *Ba. quintana* (bqtR), *Ehrlichia* spp. (16S), *E. ruminatum* (dsb), *Hepatozoon* spp. (18S), *T. gondii*, *B. microti* (CCTeta), *B. bigemina* (ARN 18S), *B. vogeli* (hsp70), *B. caballi* (rap1), *B. bovis* (CCTeta), *B. ovis* (ARN18S), *B. divergens* (hsp70), *T. equi* (Ema1), *T. annulata* (ARN 18S), *Leishmania* spp. (hsp70), *L. infantum* (ITS), and African swine fever virus (Vp72). The primers and probes for these pathogens selected from the literature were designed by Grech-Angelini et al., Gondard et al. and Michelet et al. [33,95,96]. The primer and probe sets created for this study are listed in Table 3. 

### 4.4. DNA Amplification and Microfluidic Real-Time PCR

Prior to the microfluidic real-time PCR, DNA from each sample for all targeted pathogens was amplified by simple PCR for better detection. The primers used were the same as those used for the microfluidic real-time PCR, except for *Escherichia coli*, which was not amplified.

The target vector-borne pathogens were detected using a BioMarkTM real-time PCR system (Fluidigm, South San Francisco, CA, USA) for high-throughput microfluidic real-time PCR amplification using 48.48 dynamic arrays (Fluidigm). Fluidigm chips can be used to perform 2304 real-time PCR reactions thanks to 48 PCR mixes and 48 samples placed into individual wells, prior to transfer into individual chambers for the reaction. The thermal cycling conditions were 50 °C for 2 min, 95 °C for 10 min, and 40 cycles at 95 °C for 15 s and 60 °C for 1 min. One negative water control, one inhibitory molecule control (*Escherichia coli* EDL933 strain), and one DNA extraction control (animal targeting primers) were added to each chip. Specific primers and probes of *E. coli* were used.

### 4.5. Confirmation of Pathogen Detection

Confirmation of pathogen detection was performed by nested PCR or real-time PCR. As for the assay design, most of the primers were selected from the literature if they fit into the confirmation design. The positive sequences after gel migration from the nested PCR products were sent to sequencing at Eurofins MWG Operon (Germany) and assembled using BioEdit software (Ibis Biosciences, version 7.2.5, Carlsbad, CA, USA). An online BLAST (NCBI; National Center for Biotechnology Information) was used to identify the sequenced organisms. All the target genes and primer sequences from the literature were described in the studies by Gondard et al. and Michelet et al. [95,96]. The primer/probe sets used for confirmation of detection in this study are listed in Table 4.

## Figures and Tables

**Table 1:** Tick-borne pathogen distribution in wild boar and their ticks in the Corsican wetlands.

Municipalities (No)	Sampled Wild Boar/Positive	*D. marginatus*	*Hy. marginatum*	*Hy. scupense*	*Rh. sanguineus* s.l.	Total Ticks
-	Total/Positive (A/B/R/Ra/Rs)	Total/Positive (A/B/R/Ra/Rs)	Total/Positive (A/B/R/Ra/Rs)	Total/Positive (A/B/R/Ra/Rs)	Total/Positive (A/B/R/Ra/Rs)	Total/Positive (A/B/R/Ra/Rs)
Aléria (1)	73/0	47/39 (Rs)	-	-	-	47/39 (Rs)
Appietto (2)	2/0	-	-	-	-	-
Bonifacio (3)	3/0	-	-	-	-	-
Chiatra (4)	11/3 (B)	24/1 (R), 24 (Rs)	2/1 (Ra)	-	1/1 (Ra)	27/1 (R), 2 (Ra), 24 (Rs)
Ersa (5)	3/0	5/1 (A), 4 (Rs)	-	-	-	5/1 (A), 4 (Rs)
Ghisonaccia (6)	10/0	-	-	-	-	-
Linguizetta (7)	11/0	4/1 (Rs)	-	1/1 (Ra)	-	5/1 (Rs), 1(Ra)
Luciana (8)	1/0	-	-	-	-	-
Penta di casinca (9)	2/0	8/4 (Rs)	-	-	-	8/4 (Rs)
Pianottoli-Caldarello (10)	1/0	-	-	-	-	-
Prunelli-di-Casaconi (11)	2/0	-	-	-	-	-
Solaro (12)	5/0	7/1 (Ra), 1 (Rs)	-	-	-	7/1 (Ra), 1 (Rs)
Taglio-Isolaccio (13)	2/0	5/1 (R), 3 (Rs)	-	-	-	5/1 (R), 3 (Rs)
Tallone (14)	20/0	8/1 (R), 2 (Ra), 4 (Rs)	-	-	-	8/1 (R), 2 (Ra), 4 (Rs)
Vescovato (15)	3/0	-	-	-	-	-
Ventiseri (16)	7/0	1/1	-	-	-	1
Volpajola (17)	2/0	-	-	-	-	-
Total/Positive	158/3 (B)	109/1 (A), 3 (R), 3 (Ra), 80 (Rs)	2/1 (Ra)	1/1 (Ra)	1/1 (Ra)	113/1 (A), 3 (R), 6 (Ra), 80 (Rs)

A: *Anaplasma* spp., B: *Babesia* spp., R: *Rickettsia* spp., Ra: *R. aeschlimannii*, Rs: *R. slovaca*.

**Table 3:** List of pathogens, targets and primer/probe sets created.

Pathogen	Name	Sequence	Length (Nucleotide)	Genes	Genes References
Aujeszky’s virus	Aujv_gp50_F	CTTTATCGAGTACGCCGACTG	225	*gp50*	Y14834.1
	Av_gp50_R	AACGGGCACTCTTGCCCC			
	Av_gp50_P	CAGATCTTTGGGCGCTGCCGGC			
*Ch. psitacci*	Chl_psi_16S-23S_F	ACGCCGTGAATACGTTCCC	214	*16S-23S rRNA*	U68450.1
	Chl_psi_16S-23S_R	AGTCAAACCGTCCTAAGACAG			
	Chl_psi_16S-23S_P	CCTTGTACACACCGCCCGTCACATC			
*Haemoproteus* spp.	Hae_cytB_F	ATATGCATGCTACTGGTGCTAC	240	*cytochrome B*	AF465579.1
	Hae_cytB_R	CAAATCCATGAAACAAGTCCAGG			
	Hae_cytB_P	CGGTTGCACCCCAGAAACTCATTGAC			
*Leptospira* spp.	Lep_Lipl32_F	CTCTATGTTTGGATTCCTGCC	158	*LipL32*	MK541891.1
	Lep_Lipl32_R	CCAAGTATCAAACCAATGTGGC			
	Lep_Lipl32_P	ATTGATTTTTCTTCTGGGGTAGCCGCTTTG			
*Leucocytozoon* spp.	Leu_cytB_F	GGGTTATGTCTTACCATGGGG	177	*cytochrome B*	KF717066.1
	Leu_cytB_R	AATTGCTAGTGCTACGAATGGG			
	Leu_cytB_P	AAATGAGTTTTTGGGGAGCAACCGTTATTAC			
*Plasmodium* spp.	Pla_ssrRNA_F	ATATAGAAACTGCGAACGGCTC	339	*ssrRNA*	MK650620.1
	Pla_ssrRNA_R	TTTCTCAGGCTCCCTCTCC			
	Pla_ssrRNA_P	CTCTAATTCCCCGTTACCCGTCATAGC			
*R. monacensis*	Ric_mon_F	CTCGGTGCCGGTACTTTAAAC	192	*ompB*	KU961543.1
	Ric_mon_R	GAGCACCGCCAATAAGAGC			
	Ric_mon_P	AGTGCCGATGCAAATACTCCGGTGAC			
*Trypanosoma* spp.	Try_18SRNA_F	GTAATTCCAGCTCCAAAAGCG	178	*18SRNA*	EU596263.1
	Try_18SRNA_R	TCAGGAAGGAACCACTCCC			
	Try_18SRNA_P	ACCTCAAGGGCATGGGTCACCAATCC			

**Table 4:** List of pathogens, targets and primer/probe sets used for confirmation.

Pathogen	Name	Sequence	Length (Nucleotide)	Genes	Genes References
Aujeszky’s virus	Aujv_gp50_F2	AACATCCTCACCGACTTCATG	158	*gp50*	Y14834.1
	Av_gp50_R2	CTGGTAGAACGGCGTCAGG			
	Av_gp50_P2	AATCGCATCACGTCCACGCCCCC			
*Ch. psitacci*	Chl_psi_16S-23S_F2	AGTAATCTTCGGCGAGCTGG	177	*16S-23S rRNA*	U68450.1
	Chl_psi_16S-23S_R2	CGCTACTTAGGGAATCTCTTT G			
	Chl_psi_16S-23S_P2	TATAAAGCTATGACCCGGAGGTCTCCG			
*Haemoproteus* spp.	Hae_cytB_F2	CCTTGGGGTCAAATGAGTTTC	231	*cytochrome B*	AF465579.1
	Hae_cytB_R2	AAGCCGTATCATATCCTAAAGG			
	Hae_cytB_P2	CCTGGACTTGTTTCATGGATTTGTGGAGG			
*Leucocytozoon* spp.	Leu_cytB_F2	GAGTTTCTGGGGAGCTACTG	197	*cytochrome B*	KU842391.1
	Leu_cytB_R2	GGATTAGTGCTACCTTGAATATG			
	Leu_cytB_P2	TGAATAAATACAATTGCTAGTGCTACGAATGG			
*Plasmodium* spp.	Pla_ssrRNA_F2	TCGAGTTTCTGACCTATCAGC	264	*ssrRNA*	MK650620.1
	Pla_ssrRNA_R2	AGACTTGCCCTCCAATTGTTA C			
	Pla_ssrRNA_P2	TGGCCTTGCATTGTTATTTCTTGTCACTACC			
*Trypanosoma* spp.	Try_18SRNA_F2	CAACACGGGGAACTTTACCAG	141	*18SRNA*	EU596263.1
	Try_18SRNA_R2	ATCCTACTGGGCAGCTTGG			
	Try_18SRNA_P2	CAGGGTGAGGATTGACAGATTGAGTGTTC			
